# Effect of Different Graft Material Consistencies in the Treatment of Minimal Bone Dehiscence: A Retrospective Pilot Study

**DOI:** 10.3390/dj12070198

**Published:** 2024-06-27

**Authors:** Maria Menini, Luigi Canullo, Roberta Iacono, Alessio Triestino, Vito Carlo Alberto Caponio, Paolo Savadori, Paolo Pesce, Andrea Pedetta, Fabrizio Guerra

**Affiliations:** 1 Department of Surgical Sciences (DISC), University of Genoa, Largo R. Benzi 10, 16132 Genoa, Italy; maria.menini@unige.it (M.M.); luigi.canullo@unige.it (L.C.); paolo.pesce@unige.it (P.P.); 2 Department of Oral and Maxillo-Facial Sciences, “Sapienza” University of Rome, Via Caserta 6, 00161 Rome, Italy; iacono.1636694@studenti.uniroma1.it (R.I.); fabrizio.guerra@uniroma1.it (F.G.); 3 Private Practice, Via Del Tritone 169, 00187 Rome, Italy; alessiotriestino@gmail.com; 4 Department of Clinical and Experimental Medicine, University of Foggia, Viale Pinto 1, 7100 Foggia, Italy; vitocarlo.caponio@unifg.it; 5 Department of Biomedical, Surgical, and Dental Sciences, University of Milan, Via Della Commenda, 10/12, 20122 Milan, Italy; 6 IRCCS Fondazione Cà Granda IRCCS Ospedale Maggiore Policlinico, 20122 Milan, Italy; 7 Private Practice, Via Colledoro 41, 00034 Colleferro, Italy; andrea.pedetta@gmail.com

**Keywords:** guided bone regeneration (GBR), bone grafts, volumetric tissue changes, implant insertion, collagen membrane

## Abstract

Among different therapeutic strategies proposed in the case of bone volume deficit, guided bone regeneration (GBR) is a consolidated surgical procedure. The objective of this study is to retrospectively evaluate the behavior of two bone grafts with different consistencies in the GBR procedure by measuring the volumetric tissue changes 1 year after surgery. For this retrospective analysis, 25 cases of GBR with simultaneous implant insertion were selected. A total of 13 were grafted with a porcine cortico-cancellous bone mix (CCBM group), and 12 were grafted with a pre-hydrated granulated cortico-cancellous bone mix of porcine origin blended with 20% TSV gel (Collagenated-CCBM). A collagen membrane was fixed to cover the bone defect. A total of 42 implants were placed with computer-guided surgery. Preoperative and 12-month postoperative digital impressions were used to evaluate dimensional changes. Student’s t-test used for independent samples showed no statistically significant differences between the integrated distance (*p* = 0.995) and mean distance (*p* = 0.734). The mean integrated distance in the CCBM group was 41.80 (SD. 101.18) compared to a mean of 42.04 (SD. 66.71) in the Collagenated-CCBM group. Given the limitations of this study, in patients with peri-implant bone dehiscence, simple heterologous and collagenated heterologous cortico-cancellous bone grafts are suitable for filling the bone defect to promote bone regeneration, although further studies are needed.

## 1. Introduction

Implant therapy stands as a well-established solution for the rehabilitation of edentulous areas, yet it poses a significant challenge for clinicians dealing with atrophic upper and lower jaws. The selection of appropriate treatment options to replace a missing tooth is intricately linked to the condition of the residual alveolar ridge [1]. Unfavorable situations, such as inadequate alveolar ridge width (knife-edge ridge) or insufficient thickness and height (flattened alveolar ridge), classified as class IV and V according to the Cawood and Howell classification [2], present difficulties in achieving optimal implant placement. In such scenarios, ensuring a correct position for implants and guaranteeing a favorable long-term prognosis and aesthetic outcome become challenging, leading to the proposal of various therapeutic strategies [3,4,5].

One such strategy is guided bone regeneration (GBR), a surgical procedure utilizing a membrane as a space maintainer to foster new bone formation [6]. Depending on the extent of the bone defect, GBR can be performed simultaneously with implant placement or in two separate surgical phases, with the first phase involving GBR surgery and the second comprising implant surgery.

The rationale for employing membranes is grounded in ‘the principle of compartmental wound healing’ [7]. Acting as a barrier, the membrane prevents the colonization of the wound by unwanted cells (epithelial cells), facilitating the proliferation of desired cells capable of proper regeneration, which exhibit a slower turnover. Membranes play a pivotal role in promoting bone regeneration by preventing epithelial migration and stabilizing the blood clot in the bone defect [8].

The GBR technique has undergone refinement over the past two decades, resulting in numerous strategies aimed at improving the efficacy and predictability of the surgical procedure. Various generations of membranes have been developed, with selection criteria based on their space-making ability, physical and mechanical properties, handling, and biocompatibility [9,10].

While membranes actively contribute to promoting bone regeneration, they are not the sole biomaterials employed in GBR techniques. Often, the peri-implant bone deficit is addressed by applying bone grafts to enhance regeneration and prevent membrane collapse onto the residual bone due to soft tissue pressure [11,12]. Autologous bone graft, currently considered the gold standard, possesses excellent osteogenic, osteoinductive, and osteoconductive properties [13]. However, its limitations, including harvesting constraints, the need for a second surgical site, prolonged surgical times, and increased post-operative discomfort for the patient, necessitate the exploration of alternative options [14,15]. Moreover, pure autologous bone grafts undergo resorption over time. To mitigate this phenomenon, they can be combined with or substituted by other graft materials.

To overcome these limitations, heterologous bone grafts are frequently employed, either alone or combined with autologous grafts, showing favorable results in bone augmentation procedures. Research efforts have been directed toward bone graft substitutes, including allografts, xenografts, and synthetic (alloplastic) grafts [16,17,18].

Bovine, porcine, and equine xenografts are widely utilized due to their similar morphology to human bone, high osteoconductive ability, and biocompatibility [19]. This category also includes collagenated heterologous bone grafts enriched with collagen to enhance osteoconductivity and act as a scaffold for bone formation [20,21]. Notably, biomaterials with collagen molecules embedded into bone granules have demonstrated physicochemical properties similar to native human bone, proving effective in various surgical procedures [22,23,24,25,26]. In this study, a recent heterologous bone substitute with collagen molecules embedded into bone granules (OsteoBiol^®^ GTO^®^) was considered.

The primary objective of this study is to retrospectively evaluate the success of the GBR horizontal technique in treating vestibular Class 1 dehiscence (Tinti et al. Classification [27]) using two different bone graft substitutes. In the first group, we used cortico-cancellous heterologous bone graft in association with a collagen membrane. In the second group, we used collagenated heterologous cortico-cancellous bone graft in association with a collagen membrane.

## 2. Material and Methods

This retrospective analysis included 25 subjects consecutively treated between September 2020 and November 2022 in a private dental practice in Rome. One surgeon (L.C.) performed all the surgical procedures.

Patients requiring implant placement in the lower and upper jaw with simultaneous horizontal bone augmentation for treating vestibular Class 1 dehiscence, according to Tinti et al.’s [27] classification, were included in the study. General inclusion and exclusion criteria are listed in Table 1.

Patients were divided into two groups according to the graft material used:

- CCBM group: Guided bone regeneration performed with porcine cortico-cancellous bone mix (OsteoBiol^®^ Apatos^®^ Mix, Tecnoss^®^, Giaveno, Italy) and a collagen membrane (OsteoBiol^®^ Evolution, Tecnoss^®^, Giaveno, Italy)—APATOS Group (13 patients).
- Collagenated-CCBM: Guided bone regeneration performed with a pre-hydrated granulated cortico-cancellous bone mix of porcine origin blended with 20% TSV gel, which is a mixture of heterologous type I and type III collagen gel with polyunsaturated fatty acids and a biocompatible synthetic copolymer diluted in aqueous solution. (OsteoBiol^®^ GTO^®^) and a collagen membrane (OsteoBiol^®^ Evolution)—GTO Group (12 patients).

All patients were treated in accordance with the World Medical Association’s Declaration of Helsinki and Good Clinical Practice Guidelines. The local Ethics Committee of the University of Genoa (CERA protocol no. 2023.83) approved the conduct of the study. After ethical approval, all patients signed an informed consent allowing for the use of their data for scientific purposes. Patient data were anonymized.

### 2.1. Preoperative Procedure

Before surgery, all patients underwent a clinical assessment and CBCT evaluation. A professional oral hygiene session and an intraoral digital scan were performed. All patients received personalized instructions for home oral hygiene.

Digital scanning (CS 3600 intraoral scanner, Carestream, Rochester, NY 14608, USA) and 3D radiographic scanning (CS 8100 Cone Beam, Carestream, Rochester, NY 14608, US) were performed. Then, DICOM and STL files were matched and used to plan the surgery virtually (RealGUIDE, 3diemme, Cantù, CO, Italy).

Following the planning, a surgical guide was produced to achieve a correct implant placement.

The preoperative digital scan was used as reference data for volumetric analysis.

### 2.2. Surgical Procedure

The surgery was performed under local anesthesia (mepivacaine with adrenaline 1:100,000). A full-thickness flap was elevated, and a surgical guide was inserted. The planned implant site preparation was performed in the exact site indicated by the surgical guide using a specific drill sequence according to the manufacturer’s instructions. After implant placement (Multineo CS, Alphabio, Israel), the bone defect was measured with a PCP15 periodontal probe, and the regenerative site was decorticated to increase blood supply. An OsteoBiol^®^ Evolution membrane, fixed with titanium osteosynthesis screws, was used to cover the defects. The site was then filled either with a graft material consisting of pre-hydrated collagenated heterologous cortico-cancellous bone (OsteoBiol^®^ GTO^®^) using an applicator syringe (Collagenated-CCBM group) or with a graft material consisting of cortico-cancellous bone (OsteoBiol^®^ Apatos^®^) mixed with the patient’s blood in a sterile dappen (CCBM group). The graft material was placed to overfill the defect between the bone and the membrane and was carefully condensed at each stage.

Finally, the mucosa was sutured using a coronally advanced flap technique with modified mattress sutures with 6.0 PGLA (Monofast, Medipac, Athens, Greece) to achieve first-intention healing.

### 2.3. Postoperative Procedure

Antibiotic therapy was prescribed with 1 g of amoxicillin every 8 h for 4 days from the evening before surgery; postoperative pain management was achieved with 600 mg of Ibuprofen orally every 12 h for 3 days and with cold application for the first 2 days [28]. Rinses with 0.12% chlorhexidine gluconate were prescribed for 14 days. Sutures were removed after 14 days. Postoperative CBCT scans have not been performed.

### 2.4. Prosthetic Procedure

Three months after surgery, the implants were uncovered through a minimal midcrestal incision, and a healing abutment was placed. After an additional 4 weeks, all implants were functionally loaded with screw-retained zirconia crowns. Twelve months after surgery, a postoperative intraoral scan was performed in order to assess the volumetrical changes in comparison with the preoperative intraoral scan.

The Ethics Committee did not authorize the implementation of a postoperative CBCT, and consequently, no postoperative CBCT was performed. In addition, the Ethics Committee has approved the use of a 12-month postoperative scan to indirectly assess volumetric changes with a 1-year follow-up period by the analysis of the superimposition of preoperative intraoral scan and 1-year postoperative intraoral scan.

### 2.5. Volumetric Outcomes

The present clinical study used three-dimensional computer-aided design (CAD) analysis software to measure the hard and soft tissue response after bone regeneration surgery (GOM Inspect, GOM GmbH, Braunschweig, Germany).

A preoperative and postoperative intraoral scan was performed for each patient using an intraoral scanner (CS 3600 Carestream, Rochester, NY, USA).

The preoperative model was imported into the GOM Inspect 2018 software as a nominal CAD value, and the postoperative model was imported as a current mesh value, as Seidel et al. [29] reported.

As suggested by Schmitt et al. [30], to obtain a correct superimposition of the models, they were first superimposed using 3-point superimposition (association of three prominent structures of the actual value to the corresponding structures in the mesh value) and then aligned using the “local best-fit function” (association of individual structures within the scan, e.g., the occlusal plane of adjacent teeth). This allowed the models to be accurately superimposed.

For each regenerated site, a region of interest (ROI) was individually selected on the vestibular portion of the gingival tissue, not exceeding the mucogingival line.

A surface comparison analysis was performed using the GOM Inspect software to calculate the volumetric results in each ROI (region of interest), as reported by Schmitt et al. [30]. The surface comparison analysis provides the surface deviation between the preoperative (CAD value) and the postoperative scan (actual value). In principle, the surface comparison calculates a deviation of the actual value (mesh) from the nominal value (CAD) for each point.

**Figure 1:**
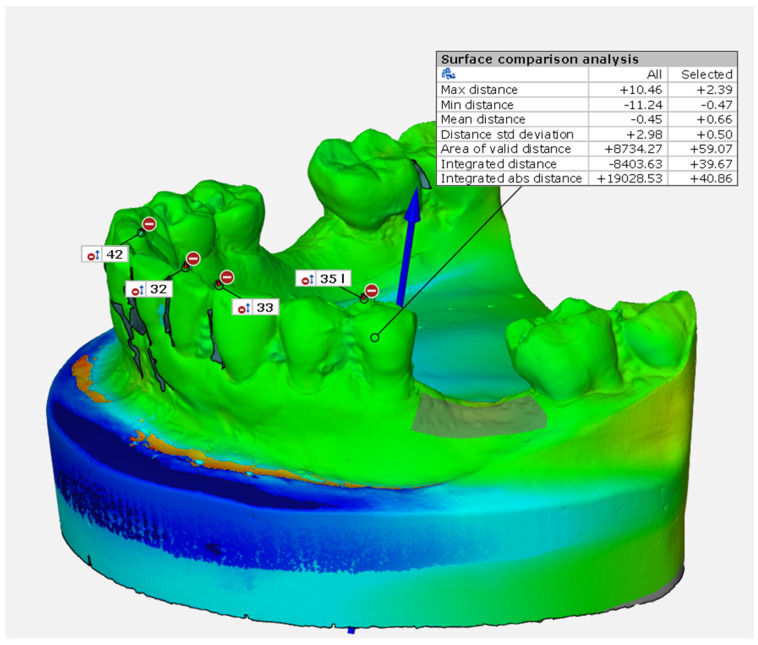
Surface comparison analysis.

From a geometric point of view, a surface comparison analysis is geometrically identical to the mesh surface (Figure 1).

The colored parts describe the difference between the nominal data (the preoperative scan) and the actual data (the postoperative scan). From a clinical point of view, the surface comparison analysis represents the variation between the preoperative and the postoperative scans. Therefore, it can be considered tissue gain or loss after regenerative procedures.

**Figure 2:**
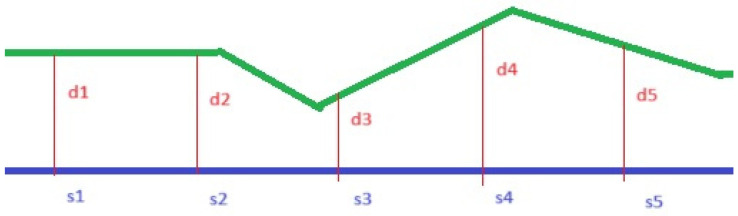
Schematic calculation of the deviation (2D) area inside an ROI. The area under the curve is the “integrated distance”, which describes the tissue gain or loss after regenerative procedures.

It is possible to calculate the area of the deviation between the nominal value (CAD—preoperative scan) and the actual value (mesh—postoperative scan) in a specific ROI by performing the integral calculation (area under the curve). The calculation is performed on all surfaces in 3D inside the ROI. The following schematic sketch is shown in 2D and describes the calculation of the deviation area inside an ROI. (Figure 2).

The area of the deviation is calculated with the following formula, and it is called “integrated distance” (ID):

$$Integrated\;Distance=\sum \left(d_{i}x\;s_{i}\right)$$

The integrated distance describes the volume increase in mm^3^ in the buccal part of the augmented region; consequently, it was the primary outcome of this study. Secondary outcomes were calculated to investigate the extent of the difference between the pre- and postoperative scans.

- Mean distance (MeanD) in mm in the augmented region. The mean distance describes the arithmetic mean deviation (mm) of all surface comparison points from preoperative and postoperative scans. It describes the medium extent of the regenerated area in each augmented region.
- Maximum distance (MaxD) in mm in the augmented region. The maximum distance describes the maximum deviation (mm) of the surface comparison between the preoperative and the postoperative scans. It describes the maximum extent of the regenerated area in each augmented region.
- Minimum distance (MinD) in mm in the augmented region. The minimum distance describes the minimum deviation of the surface comparison between the preoperative and postoperative scans. It describes the minimum extent of the regenerated area in each augmented region.

### 2.6. Statistical Analysis

The clinical characteristics of patients were examined between the CCBM group and the Collagenated-CCBM group to account for heterogeneity between groups using Fisher’s exact test. The Shapiro–Wilk test was used to examine the normal distribution of variables for all linear variables.

While a non-normal distribution was observed for most of the outcomes, the integrated distance and mean distance followed a normal distribution (Shapiro–Wilk *p*-value 0.220 and 0.207, respectively). In this context, Student’s independent samples t-test was used to examine the mean differences between the CCBM group and Collagenated-CCBM. A two-way ANOVA test was performed to determine whether the primary outcome, such as the integrated distance, was influenced by both the type of treatment group and the anatomical subsite (upper versus lower maxilla). Based on previous considerations, the Mann–Whitney test was used to examine the mean differences between groups for non-parametric variables.

## 3. Results

For this retrospective study, 25 patients were analyzed; 13 were included in the CCBM group and 12 were included in the Collagenated-CCBM group. A total of 42 implants were placed simultaneously with the GBR, and no implants were lost before or after loading. The mean age did not differ between the two groups (*p*-value = 0.698), nor did the patients differ in clinical characteristics. Fisher’s exact test showed no statistically significant *p*-values when taking into account sex (*p*-value = 0.226), treatment in the upper or lower maxilla (*p*-value = 0.695), and tooth area, such as incisor–canine versus premolar–molar area (*p*-value = 0.999) (Table 2).

Student’s t-test for independent samples showed no statistically significant differences between the integrated and mean distance (Table 3). Patients in the CCBM group had a mean integrated distance of 41.80 (SD. 101.18) versus a mean of 42.04 (SD. 66.71) in the Collagenated-CCBM group (Table 3).

When considering treatment groups and anatomical subsites, the simple primary effect analysis confirmed the previous results, showing that the different treatment groups did not affect the mean integrated distance (*p*-value = 0.685), nor did the different anatomical subsites (*p*-value = 0.294) (Table 4).

**Figure 3:**
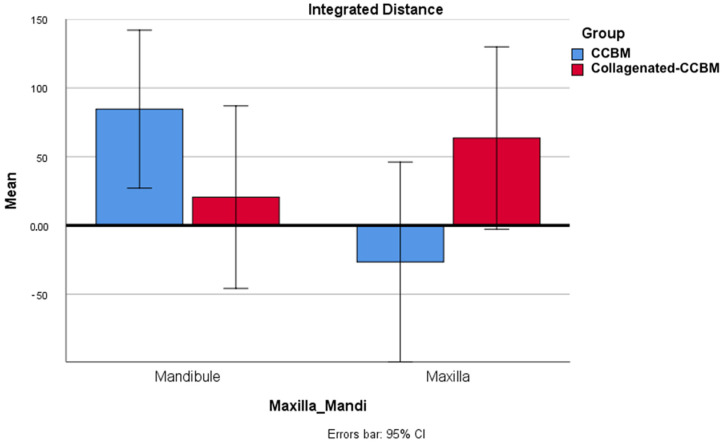
Combined effect in the two-way ANOVA revealing that the mean integrated distance in the upper maxilla was reduced in the CCBM group compared to both the CCBM treatment in the lower maxilla and Collagenated-CCBM treatment in the upper maxilla.

The combined effect in the two-way ANOVA revealed a strong interaction between the type of treatment performed in either the upper or lower maxilla (two-way ANOVA *p*-value = 0.024). The integrated distance mean in the upper maxilla was reduced in the CCBM group compared to the CCBM treatment in the lower maxilla and the Collagenated-CCBM treatment in the upper maxilla (Figure 3).

Student’s t-test for independent samples showed no statistically significant differences between the integrated and mean distance (Table 2).

Similarly, the maximum and minimum distance means did not differ between the CCBM and Collagenated-CCBM groups (Mann–Whitney *p*-values 0.406 and 0.852, respectively). Similar results were obtained when looking at the area of the valid distance (*p*-value = 0.110). However, the distance standard deviation and the integrated abs distance differed significantly between groups (*p*-values 0.019 and 0.022, respectively). The mean distance standard deviation was 2.34 (SD 0.34) in the CCBM group and 1.18 (SD 0.23) in the Collagenated-CCBM. A higher mean value was also found for the integrated abs distance in the CCBM group (114.84, SD 19.9) than in the Collagenated-CCBM group (75.35, SD 13.2).

## 4. Discussion

The presence of collagen in biomaterials, particularly highlighted in our study with OsteoBiol^®^ GTO^®^, has been consistently associated with positive outcomes. Collagen is crucial in platelet aggregation, neovascularization, bone marrow stem cell differentiation, and osteogenic cell recruitment [31,32,33,34].

The bone grafts utilized in this study have been previously evaluated in various surgical applications, such as sinus lift and alveolar ridge preservation, by other authors [31,32,33,34,35]. For instance, Comuzzi and colleagues reported lower graft shrinkage for collagenated biomaterials with a diameter of up to 300 microns in crestal access sinus lift compared to those with a diameter exceeding 500 microns, such as OsteoBiol^®^ GTO^®^ [36,37]. Barone et al. demonstrated superior dimensional changes in porcine cortical bone after tooth extraction, with a notable decrease in volume shrinkage for sockets grafted with OsteoBiol^®^ mp3^®^, a pre-hydrated collagenated cortical cancellous bone mix [38,39].

This kind of bone substitute was prepared with a low-temperature production process that does not denature collagen molecules and leaves them embedded into xenogenic bone granules [23]. Bone turnover and volumetric changes are attributed to osteoclasts and osteoblasts that require O_2_ and nutrients to carry out this process. Nutrients and gases are provided by blood vessels or capillaries, and VEGF was demonstrated to be a master gene for angiogenesis [40,41]. In 2016, Rombouts and collaborators proved that collagenated graft granules, but not anorganic bovine bone (ABB), significantly enhanced VEGF secretion in human periodontal ligament (PDL) cells. The higher amount of VEGF secreted led to a significantly higher proliferation of endothelial cells and capillaries with larger diameters [37].

Furthermore, mesenchymal stem cells (MSCs) play a fundamental role in bone regeneration [39]. When comparing collagenated graft materials to ABB, Jeanneau demonstrated that the former significantly increased the secretion of the protein C5a from injured human PDL cells, its interaction with the C5a receptor (C5aR) placed on the cell surface of MSCs, its subsequent phosphorylation, and the consequent migration of MSCs towards injured human PDL cells.

Finally, collagenated biomaterials were reported to promote human periodontal ligament stem cell (PDLSC) osteogenic differentiation, as witnessed by the fact that collagen type I secreted and calcium deposited by PDLSCs seeded onto OsteoBiol^®^ Sp-Block and placed into an osteogenic growth medium were higher than the control group (osteogenic growth medium only) [42].

Additional information was provided by Di Tinco et al. The mentioned study investigated the osteogenic differentiation, stem cell properties, and inflammatory properties of neural crest-derived stem cells isolated from human dental pulp (hDPSCs) to differently composed collagenated grafts than traditional, non-collagenated, heterologous grafts [43]. The authors, in fact, report that both groups of biomaterials did not affect the stemness potential of hDPSCs nor induce any immune response. However, when analyzing the osteogenic differentiation of hDPSCs, it was found that differentiated hDPSCs (diff-hDPSCs)-exposed collagenated grafts showed a high ALP activity, with the higher presented by OsteoBiol^®^ GTO^®^. Conversely, non-collagenated graft-treated diff-hDPSCs showed a lower ALP activity than diff-hDPSCs alone. The authors of the study conclude that OsteoBiol^®^ GTO^®^ and OsteoBiol^®^ Gen-Os^®^ are preferable from a clinical standpoint because they exert both a mechanical function and an osteogenic-promoting function.

This study pioneers the use of these materials in lateral bone augmentation, resulting in increased bone volume 12 months post-surgery.

The volumetric changes in hard and soft tissues were meticulously assessed by superimposing preoperatively and postoperatively. STL files are a widely accepted method in the literature for studying dimensional changes without using invasive procedures [44,45,46].

The use of heterologous bone as an alternative to autologous bone increased bone volume and avoided the need for a second surgical site for bone harvesting, minimizing surgical complications and reducing patient morbidity. No intraoperative or postoperative complications were reported, affirming the safety and efficacy of the GBR procedure. The resorbable collagen membrane further contributed to less invasive GBR surgical procedures, aligning with the findings from other studies [47,48].

After 12 months of functional loading, the survival rate was 100%, and all implants had adequate osseointegration. This underscores the predictability and safety of GBR with the simultaneous implant approach. However, it is noteworthy that our results differ from the implant survival rates reported by Danlos et al., who recorded rates of 87% to 95%. This disparity may indicate the need for further investigation into the comparative effectiveness of one-stage versus two-stage GBR techniques [49].

The integration of computer-guided surgery proved instrumental in eliminating intraoperative complications and enabled the precise assessment of required bone augmentation through planning software. Additionally, the utilization of OsteoBiol^®^ GTO^®^, a collagen-based sticky bone graft substitute, facilitated the efficient filling of bone defects, reducing surgical times.

While this study contributes valuable insights, certain limitations must be acknowledged. These include the small sample size, a relatively short follow-up period, the absence of histological data, and a lack of postoperative three-dimensional radiographic evaluations. The data from surface scanning may be influenced by inflammatory states, leading to the thickening of the oral mucosa, thus potentially providing volumetric data that are not representative of the volume of newly formed bone. On the other hand, the procedure described by Schmitt et al. [30] significantly reduces this possibility and the corresponding alteration of data. However, for intellectual honesty, the authors have considered it appropriate to mention this possibility. Prospective studies and randomized controlled clinical trials are warranted to provide more in-depth analyses of the behavior of these materials in GBR procedures and to establish their long-term efficacy and safety.

## 5. Conclusions

Beyond the limitations of the present study, GBR with OsteoBiol^®^ Apatos^®^ Mix and OsteoBiol^®^ GTO^®^ produced horizontal bone ridge augmentation with simultaneous implant placement. Despite the differences in consistency, both bone graft substitutes were found to be effective and suitable for filling the peri-implant bone dehiscence and promoting new bone formation.

## Figures and Tables

**Table 1:** General inclusion and exclusion criteria.

General Inclusion Criteria	General Exclusion Criteria
Male or female ≥ 18 years old	Smoker > 10 cig per day, cigar equivalents or tobacco chewers
Patients willing to participate and to attend the planned follow-up visits	History of leukocyte dysfunction and deficiencies
	History of neoplastic disease requiring the use of radiation or chemotherapy
	History of renal failure
	Alcoholism or any drug abuse
	Physical handicaps that would interfere with the ability to perform adequate oral hygiene

**Table 2:** Fisher’s exact test for heterogeneity for groups showing no differences between sex (*p* = 0.226), lower or upper jaw (*p* = 0.695), and teeth area (*p* = 0.999).

Variables		CCBM Group	Collagenated-CCBM Group	*p*-Value
Sex	Male	7	3	0.226
	Female	6	9	
Jaw	Lower	8	6	0.695
	Upper	5	6	
Teeth area	Incisor–canine	3	2	0.999
	Premolar–molar	10	10	

**Table 3:** Integrated distance and mean distance in CCBM group and Collagenated-CCBM groups. Student’s t-test for independent samples showed no statistically significant differences for integrated distance (*p* = 0.995) and mean distance (*p* = 0.734).

	Group	Sample	Mean (mm^3^ for ID, mm for MD)	Std. Deviation	Standard Error of the Mean	*p*-Value
Integrated distance (ID)	CCBM	13	41.8077	101.18147	28.06269	0.995
	Collagenated-CCBM	12	42.0433	66.71674	19.25946	
Mean distance (MD)	CCBM	13	0.8400	1.05029	0.29130	0.734
	Collagenated-CCBM	12	0.6958	1.04677	0.30218	

**Table 4:** Mean integrated distance in lower maxilla and upper maxilla for both groups (CCBM and Collagenated-CCBM). The two-way ANOVA test showed that the different treatment groups (CCBM or Collagenated-CCBM) did not affect the mean integrated distance (*p* = 0.685) nor the anatomical subsite (*p* = 0.294).

Maxilla_Mandible Group					
		Mean Integrated Distance (mm^3^)	Standard Error	95% Confidence Interval	
				Lower Limit	Upper Limit
Lower maxilla	CCBM	84.560	27.631	27.098	142.022
	Collagenated-CCBM	20.568	31.906	−45.783	86.919
Upper maxilla	CCBM	−26.596	34.951	−99.280	46.088
	Collagenated-CCBM	63.518	31.906	−2.833	129.869

## Data Availability

Data available on request due to privacy and ethical restrictions.
